# Human Polymerase δ-Interacting Protein 2 (PolDIP2) Inhibits the Formation of Human Tau Oligomers and Fibrils

**DOI:** 10.3390/ijms22115768

**Published:** 2021-05-28

**Authors:** Kazutoshi Kasho, Lukas Krasauskas, Vytautas Smirnovas, Gorazd Stojkovič, Ludmilla A. Morozova-Roche, Sjoerd Wanrooij

**Affiliations:** 1 Department of Medical Biochemistry and Biophysics, Umeå University, 90 187 Umeå, Sweden; kazutoshi.kasho@umu.se (K.K.); gorazd.stojkovic@umu.se (G.S.); 2 Life Sciences Center, Institute of Biotechnology, Vilnius University, LT-10257 Vilnius, Lithuania; chemlukras@gmail.com (L.K.); vytautas.smirnovas@bti.vu.lt (V.S.)

**Keywords:** Tau, PolDIP2, amyloid

## Abstract

A central characteristic of Alzheimer’s disease (AD) and other tauopathies is the accumulation of aggregated and misfolded Tau deposits in the brain. Tau-targeting therapies for AD have been unsuccessful in patients to date. Here we show that human polymerase δ-interacting protein 2 (PolDIP2) interacts with Tau. With a set of complementary methods, including thioflavin-T-based aggregation kinetic assays, Tau oligomer-specific dot-blot analysis, and single oligomer/fibril analysis by atomic force microscopy, we demonstrate that PolDIP2 inhibits Tau aggregation and amyloid fibril growth in vitro. The identification of PolDIP2 as a potential regulator of cellular Tau aggregation should be considered for future Tau-targeting therapeutics.

## 1. Introduction

Aging-related neurodegenerative diseases are a worldwide problem with limited therapeutical solutions. Even for the most commonly diagnosed neurodegenerative diseases, such as Alzheimer’s disease (AD) and Parkinson’s disease (PD), there are no effective therapies available that would directly target the cause of the disease. Amyloid β (Aβ), α-synuclein and Tau (also known as MAPT—microtubule-associated protein Tau) are aggregation-prone proteins that play an important role in disease development [1,2,3,4]. 

Tau is a natively unstructured protein and is required for stable microtubule function. Under certain conditions, Tau is dissociated from the microtubules and self-aggregates to form soluble oligomers [4,5]. These Tau oligomers provide seeds that can lead to Tau misfolding and further aggregation into insoluble fibrils, and the development of Tau-related pathologies, or tauopathies. For these reasons, Tau is a promising target for the development of drugs that would prevent the formation of neurofibrillary lesions. In spite of its medical relevance, little is known about the mechanisms by which Tau aggregation is regulated in human cells, which hampers the development of treatments. 

Polymerase δ-interacting protein 2 (PolDIP2, also known as PDIP38) has recently been identified as a specific binding partner of Tau monomers, but not Tau oligomers or fibrils [6]. PolDIP2 is a multi-functional protein originally described as a binding partner of the nuclear DNA polymerase Pol δ and proliferating cell nuclear antigen [7,8], but previous reports have suggested that PolDIP2 has also a regulatory role in cellular Tau aggregation [6,9]. The mechanism by which PolDIP2 regulates Tau aggregation remains to be addressed.

Here, we studied Tau aggregation in the absence and presence of PolDIP2 using thioflavin T (ThT)-based aggregation assays, Tau oligomer-specific dot-blot analysis, and single oligomer/fibril analysis by atomic force microscopy (AFM). We show that PolDIP2 inhibits aggregation and amyloid fibril development of the Tau protein. 

## 2. Materials and Methods

### 2.1. Preparation of Recombinant PolDIP2 and Tau

For the purification of human PolDIP2, a DNA fragment encoding PolDIP2 amino acids 59 to 355 was cloned in the pET His_1a plasmid, obtaining a TEV-cleavable N-terminally 6xHis-tagged PolDIP2. pET His_1a-PolDIP2 was used to transform *E. coli* ArcticExpress (DE3) cells. An overnight culture was grown at 30 °C, and used for inoculation of 1.5 L terrific broth (TB) supplemented with 50 µg/mL ampicillin that was incubated at 30 °C until A_600_ 0.8. The temperature was then reduced to 12 °C and protein expression induced by addition of 0.4 mM IPTG. After 42 h of incubation, cells were collected by centrifugation at 5000× *g* for 10 min at 4 °C. Pellets were washed with ice-cold PBS and then resuspended in lysis buffer A (40 mM Tris-HCl pH 7.5, 5% glycerol, 500 mM NaCl, 1 mM DTT, 0.5% Tween 20, 1 μM pepstatin, 0.1 mM AEBSF, 1 mM DTT and 1 mM E64). Cells were lysed by pulse-sonication of cell suspensions on ice, and cleared by centrifugation at 25,000× *g* for 30 min at 4 °C. Cell lysates were supplemented with 10 mM imidazole and incubated with Ni-Sepharose excel resin (GE Healthcare, Chicago, IL, USA) for 2 h at 4 °C. The beads were washed with buffer B (40 mM Tris-HCl pH 7.5, 5% glycerol, 500 mM NaCl) with 20 mM imidazole, buffer B with 50 mM imidazole, and the proteins were eluted with buffer B containing 500 mM imidazole. Elution fractions containing PolDIP2 were incubated with TEV protease at 4 °C and dialyzed into buffer B with 20 mM imidazole. The cleaved His peptides were removed by passing the protein solution though the Ni-Sepharose column once more. Then the flow through fraction from the Ni-Sepharose column was concentrated using Vivaspin 20 (30 kDa cut-off) and further purified by gel filtration using a Superdex 200 Increase 10/300 GL column using buffer C (40 mM Tris-HCl pH 7.5, 100 mM NaCl). Protein samples were frozen in liquid N_2_ and stored at −80 °C.

For the purification of the human Tau protein (isoform 2N4R), a Champion™ pET302/NT-His plasmid carrying a DNA fragment encoding the Tau sequence fused with a SUMO protein (Supplementary Figure S1) was ordered from ThermoFisher Scientific (Waltham, MA, USA), and used to create the ULP1-cleavable N-terminally His-tagged and SUMO-tagged Tau. pET302/NT-His-SUMO-Tau were transformed in *E. coli* BL21(DE3) OneStar cells and cultured on a petri plate with LB agar supplemented with 100 µg/mL ampicillin and incubated overnight at 37 °C. A colony was inoculated into 50 mL LB supplemented with 100 µg/mL ampicillin and grown overnight at 37 °C. An overnight culture was used for 100-fold inoculation of 3.6 L ZYM-5052 autoinduction medium supplemented with 100 µg/mL ampicillin that was incubated at 37 °C until A_600_ 0.8–1.0. The temperature was then reduced to 25 °C and the culture was incubated for 12 h total. Cells were collected by centrifugation at 6000× *g* for 20 min at 4 °C. Cell pellets were homogenized in buffer A (50 mM Tris-HCl pH 8.0, 300 mM NaCl, 15 mM imidazole, 2 mM β-mercaptoethanol, EDTA-free protease inhibitor mix (ThermoFisher Scientific, Waltham, MA, USA) and lysed by sonication of cell homogenate on wet ice. The lysed cell suspension was then supplemented with 5 mM MgCl_2_ and incubated with DNAse I for 30 min with constant mixing at 4 °C. The suspension was cleared by centrifugation at 30,000× *g* for 35 min at 4 °C. The supernatant was then filtered (0.45 μm pore size syringe filter) and loaded on the BabyBio Ni-NTA column (Bio-Works, Uppsala, Sweden). The column was washed with a 2% gradient of buffer B (50 mM Tris-HCl pH 8.0, 300 mM NaCl, 500 mM imidazole, 2 mM β-mercaptoethanol), and the Sumo-Tau protein was eluted with a 20% buffer B gradient. Elution fractions containing the target protein were pooled, imidazole was removed by loading fractions onto a HiTrap Desalt column (ThermoFisher Scientific, Waltham, MA, USA) and washed with buffer C (50 mM Tris-HCl pH 7.4, 300 mM NaCl, 1 mM DTT). Desalted Sumo-Tau protein fractions were mixed with ULP1 Sumo protease (construct containing C-terminal His-tag in a pET23a vector (kind gift of prof B. Burmann), purified in a single step with Ni-NTA column) to a 1000:1 ratio and incubated for 3 h at 4 °C. Cleaved His-Sumo peptides and ULP1 protease were removed by loading the protein mixture on a HisTrap™ FF Column (ThermoFisher Scientific, Waltham, MA, USA) and washing with a buffer C and 4% buffer D (50 mM Tris-HCl pH 7.4, 300 mM NaCl, 1 mM DTT) gradient. Collected fractions containing Tau were concentrated using Amicon^®^ Ultra-15, 10 kDa (Merck, Darmstadt, Germany), and further purified by gel filtration using a Superdex 200 Tricorn 10/300 column (GE Healthcare Life Sciences, Chicago, IL, USA) with buffer E (100 mM HEPES pH 7.2, 1 mM DTT). Fractions containing the target protein were pooled and desalted again using a HiTrap Desalt column (ThermoFisher Scientific, Waltham, MA, USA) with buffer F (50 mM NH_4_HCO_3_ pH 7,8). Protein fractions were collected, lyophilized and stored at −20 °C.

### 2.2. Reaction Buffers

Reaction buffer PBS-TCEP, pH 6.7, was prepared by diluting 0.5 mM TCEP in PBS, pH 7.4, filtered through a sterile 0.22 μm pore size PES membrane filter, and stored at −80 °C. The ThT solution was prepared as described previously [10,11]. Heparin solution was freshly prepared before each experiment by dissolving dry powder of heparin sodium salt from porcine intestinal mucosa (Sigma-Aldrich, St Louis, MO, USA) in PBS-TCEP, and filtration.

### 2.3. Size Exclusion Chromatography

The mixture (500 µL) of 10 µM PolDIP2 and/or 10 µM Tau were prepared in PBS-TCEP buffer supplemented with 5.3 µM heparin, and incubated at 37 °C for 5 min. Proteins were then separated using a Superdex 200 Increase 10/300 GL column with PBS-TCEP buffer, and the fraction were analyzed by SDS-PAGE and staining using PageBlue™ Protein Staining Solution (Thermo Scientific, Waltham, MA, USA).

### 2.4. ThT-Based Tau Aggregation Assay

The reaction solution was prepared in 1.5 mL tubes at room temperature as previously described [12], and contained 15 µM Tau, 8 µM heparin, 50 µM ThT and 5 or 15 µM of PolDIP2. First, the PolDIP2 and/or Tau proteins were diluted in PBS-TCEP buffer, followed by the addition of freshly prepared heparin solution. ThT solution was added to the reaction mixture, which was then transferred (final volumes 120 µL) to a 96-Well Half-Area Microplate, Non-Binding Surface (Corning, Corning, NY, USA). The plates were sealed, placed into a Tecan Infinite F200 fluorescence microplate reader, and incubated at 37 °C with orbital shaking (3 mm amplitude and 5 s duration). ThT fluorescence was recorded every 15 min using excitation and emission filters at 440 nm and 485 nm, respectively. Each measurement was performed in 2–4 replicates. Lag time was calculated based on the 5% increase of the ThT fluorescence signal.

### 2.5. Filter Trap Assay

The reaction solution was prepared in a 1.5 mL tube at room temperature. First, Tau and/or PolDIP2 were diluted in PBS-TCEP buffer. Second, freshly prepared heparin solution was added to a final concentration of 8 µM. After centrifugation, the samples were transferred to a 96-well plate (final volumes 120 µL, one sample per each time point). The plate was sealed and placed into a Tecan Infinite F200 fluorescence microplate reader and incubated at 37 °C with orbital shaking (3 mm amplitude and 5 s duration). At each time point (4, 8, 24, 48 and 72 h), 1 µL aliquots were withdrawn from the reaction mixture and spotted on an Amersham Protran 0.45 NC nitrocellulose Western blotting membrane (GE Healthcare Life Sciences, Chicago, IL, USA). Next, the membrane was dried and blocked with 5% milk solution and incubated overnight at 4 °C with rabbit anti-Tau oligomer antibody T22 (a gift from Prof. Rakez Kayed) [13,14]. The membrane was washed with TBS-T and incubated with Goat anti-Rabbit HRP-conjugated antibody (Thermo Scientific Waltham, MA, USA) for 1 h at room temperature. After washing with TBS-T, the membrane was imaged using SuperSignal™ West Pico PLUS Chemiluminescent Substrate (Thermo Scientific Waltham, MA, USA), and analyzed using the ChemiDoc Touch Imaging System (Bio-Rad). Signal quantification was performed using ImageJ software (https://imagej.nih.gov/ij/, accessed 31 October 2020).

### 2.6. Atomic Force Microscopy (AFM)

Each sample was diluted 5 times with PBS-TCEP, and 2 µL of the solution was then spotted on the surface of freshly cleaved mica, followed by incubation for >30 s. The surface was washed 3 times with 100 µL of dH_2_O and dried overnight at room temperature. AFM imaging was performed on a PicoPlus AFM (Molecular Imaging, Tempe, AZ, USA) in tapping mode in air, using a 100-µm scanner. We used a resonance frequency in the 170–190 kHz range, a scan rate of 1 Hz and a resolution of 512 × 512 pixels. We also used a BioScope Catalyst AFM (Bruker, Billerica, MA, USA), in peak force tapping mode in air. The scan rate was 0.51 Hz, scan size 5 µm and resolution 512 × 512 pixels. Bruker MSLN and SLN cantilevers were used in all measurements [10,15].

### 2.7. Pull-Down Assay

The reaction mixture, containing 5 μM PolDIP2, 0/8/40 μM heparin and PBS-TCEP in a total volume of 50 μL, was incubated for 30 min at 37 °C with intermittent mixing. As a positive control, human polymerase γ subunit B was used in place of PolDIP2 in the assay. After the incubation, heparin sepharose beads were pelleted, and 3 μL of the supernatant, containing the unbound protein, was removed for SDS-PAGE analysis.

## 3. Results

### 3.1. PolDIP2 Interacts with Monomeric Tau

**Figure 1:**
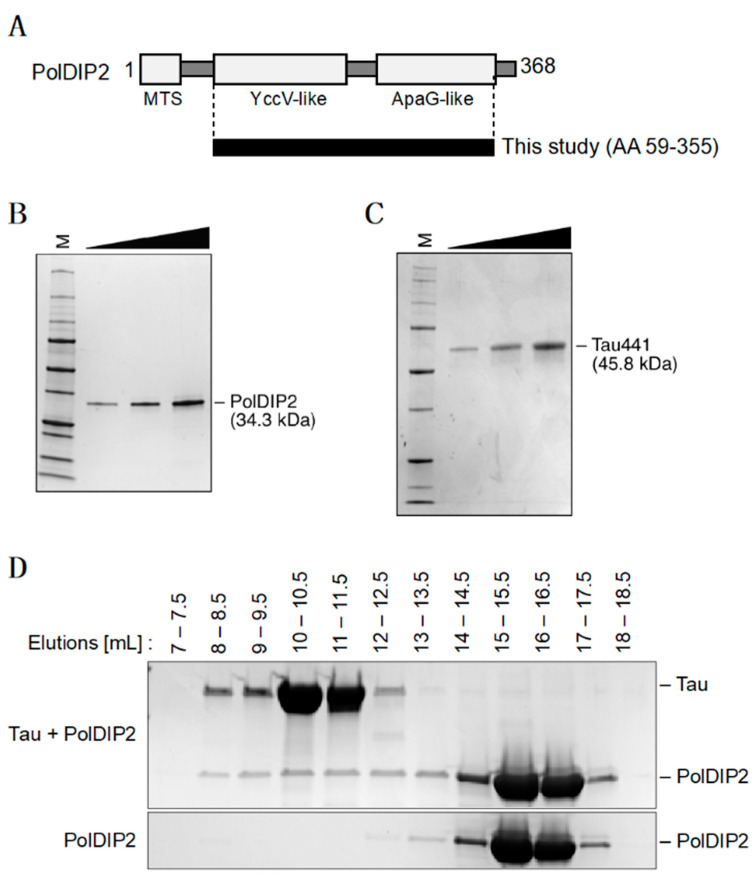
PolDIP2 interacts with Tau. (**A**) Schematic presentation of the PolDIP2 structural domains. PolDIP2 consists of an N-terminal mitochondrial targeting signal (MTS), middle YccV-like domain and C-terminal ApaG-like domain. For this study, the truncated mitochondrial form of PolDIP2 (AA 59–355) was purified. (**B**) Purification of the PolDIP2 protein. Increasing amount of PolDIP2 (150, 300 and 600 ng) loaded on SDS-PAGE gel stained with InstantBlue (Expedeon, UK). (**C**) Purification of a full-length Tau protein. Increasing amount of Tau (250, 500 and 1000 ng) loaded on SDS-PAGE gel stained with InstantBlue. (**D**) PolDIP2–Tau interaction. A total of 10 µM PolDIP2 and 10 µM Tau were incubated at 37 °C for 5 min and the mixture then analyzed by size exclusion chromatography. Proteins in the eluted fractions were separated by SDS-PAGE and stained with InstantBlue.

A cell-based screen found that human PolDIP2 interacts with Tau monomers [9], and suggested that PolDIP2 might influence the Tau aggregation process. To investigate this possibility, we purified recombinant human PolDIP2 and Tau proteins (Figure 1A–C). For our experiments, we used the PolDIP2 variant that is active inside the mitochondrial organelle (lacking the N-terminal mitochondrial targeting signal) (Figure 1A). The purified proteins did not contain a tag, were monomeric and had >95% purity (Figure 1B,C). To investigate if PolDIP2 and Tau interact in vitro, we compared size exclusion chromatography of PolDIP2 in the presence or absence of Tau (Figure 1D). In the absence of Tau, PolDIP2 was eluted as a single peak corresponding in size to the monomeric PolDIP2 (elution volume 13–17.5 mL) (Figure 1D, bottom gel). In contrast, in the presence of Tau, larger complexes could be also observed (elution volume 8–12.5 mL). A SDS-PAGE analysis showed that these larger complexes contained both PolDIP2 and Tau (Figure 1D, top gel), indicating that PolDIP2 is an interacting partner of the Tau monomer in vitro, consistent with a previous report [6].

### 3.2. PolDIP2 Inhibits Tau Amyloid Formation 

Several specific peptides or antibodies have been identified with the capability to inhibit or modulate Tau aggregation to decrease toxic oligomers or fibrils formation [16,17,18]; however, the molecular basis of cellular Tau regulation is still poorly defined. To examine if PolDIP2 can influence Tau aggregation, we employed a ThT-based protein aggregation kinetic assay and compared the measured heparin-induced Tau aggregation kinetics in the presence or absence of PolDIP2. In this assay, the ThT fluorescence signal is a measure for Tau aggregation, since ThT binds filamentous structures containing cross-β-sheets typical of amyloids, but not monomeric Tau [19,20]. Heparin contributes to the initial step of Tau fibrillation and is typically used to stimulate and analyze in vitro Tau aggregation [4].

**Figure 2:**
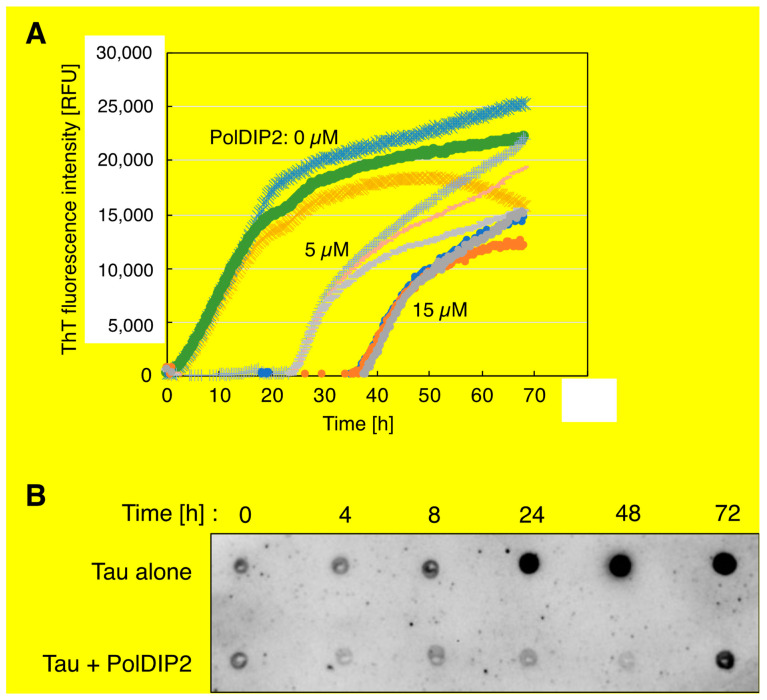
PolDIP2 inhibits Tau aggregate formation. (**A**) PolDIP2-dependent inhibition of Tau aggregation using ThT-based Tau aggregation assays. Tau (15 µM) and 8 µM of heparin were incubated at 37 °C in the absence or presence of 5 or 15 µM of PolDIP2 and 50 µM ThT. Reactions were done in 96-well microplates and fluorescent intensities were measured at 15 min intervals. (**B**) PolDIP2-dependent inhibition of Tau oligomer formation analyzed with a filter trap assay. Tau (15 µM), 0 or 5 µM of PolDIP2, and 8 µM of heparin were incubated at 37 °C. Reactions were done in 96-well microplates and paused at each time points (4, 8, 24, 48 and 72 h) to collect samples. Aliquots of 1 µL from the reactions were then spotted on nitrocellulose filter and analyzed by immunoblotting with Tau oligomer-specific antibody (T22).

As observed in Figure 2A, we detected a time-dependent increase in the ThT fluorescent signal in the three independent experiments. In the absence of PolDIP2, Tau aggregation accelerates after a lag-phase of 3.1 ± 0.1 h (Figure 2A). The ThT signal saturation was reached after about 20 h (Figure 2A). This shows that Tau alone can self-assemble in accordance with the nucleation-dependent polymerization model [4,21,22]. Interestingly, addition of PolDIP2 substantially delayed the initiation of Tau aggregation; i.e., addition of 5 and 15 µM PolDIP2 increased the lag-phase time 8- and 12-fold, respectively (25.3 ± 0 h or 38.2 ± 0.9 h) (Figure 2A). We also noticed a decrease in the maximal ThT signal intensity obtained when 5 µM or 15 µM PolDIP2 were added to the reaction (−14% and −37% maximal ThT intensity when compared with Tau alone, respectively; Figure 2A), indicating a decrease in the total quantity of aggregated species. 

We also observed that PolDIP2 by itself increased the ThT signal (Supplementary Figure S2A–C), suggesting that, under our experimental conditions, PolDIP2 forms small aggregates that interact with ThT. The starting fluorescent intensity in the ThT assay is considerably higher after addition of 5 µM (Supplementary Figure S2B) or 15 µM PolDIP2 (Supplementary Figure S2C). Subtraction of the PolDIP2 ThT background signal allowed us to study Tau specific aggregation using the ThT-based protein aggregation assay (Supplementary Figure S2A–C, Figure 2A). To ensure that PolDIP2 binds to Tau and not to heparin, which would in such a case change the free heparin concentration needed for Tau aggregation initiation, we performed an in vitro pull-down assay aimed to address this question. In contrast to polymerase γ subunit B, which bound to heparin under our reaction conditions, PolDIP2 concentration in the solution remained unchanged, showing no binding to heparin and therefore corroborating the specificity of the PolDIP2–Tau interaction (Supplementary Figure S2D).

In conclusion, the ThT experiments suggest that PolDIP2 specifically interacts with Tau and inhibits its aggregation. To validate our ThT-based assays, we in parallel used a Tau oligomer-specific (T22) antibody-based approach (Figure 2B) [13,14,23]. Incubation of Tau in the absence of PolDIP2 gives a low T22 signal after 4 h, but this signal increases at 8 h and reaches saturation levels at 24 h. This is consistent with our ThT-based assay and does confirm that after an initial lag-phase, Tau promptly forms oligomers. In the presence of PolDIP2, however, the T22-dependent signal is strongly delayed and only after an incubation for 72 h does it become detectable. This result strengthens our conclusion that PolDIP2 is able to slow down Tau aggregation. 

### 3.3. PolDIP2 Inhibits Tau Fibrillar Growth 

**Figure 3:**
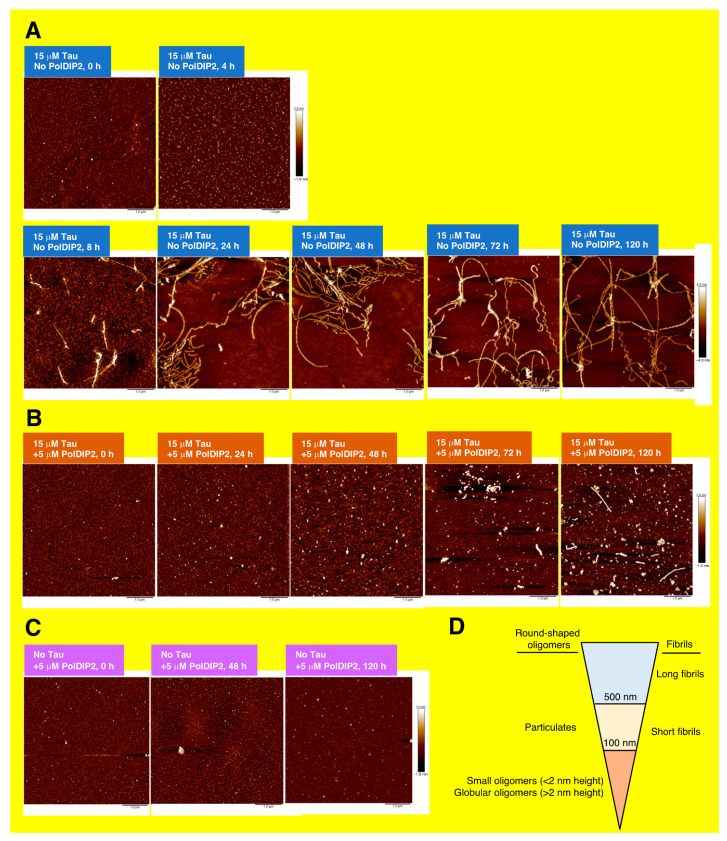
PolDIP2 inhibits Tau fibril formation. (**A**–**C**) AFM analysis of Tau fibrils and globular oligomers in the absence and presence of PolDIP2. AFM images of samples at the different indicated time points: (**A**)15 µM Tau, (**B**)15 µM Tau with 5 µM PolDIP2, and (**C**) 5 µM PolDIP2. Color scales in the *z*-axis are indicated next to each panel. The size of the scans is 5 μm × 5 μm. (**D**) A schematic description explaining the definitions used in this study to describe the AFM-observed fibrils and oligomers. We distinguish the round-shaped oligomers into “Particulates” (>100 nm diameter), “globular oligomers” (>2 nm height and <100 nm diameter) and “small oligomers” (<2 nm height and <100 nm diameter). The fibrils were sorted into “short fibrils” (100–500 nm length) and “long fibrils” (>500 nm length). The diameter was measured at half of the maximal height of the structure.

**Figure 4:**
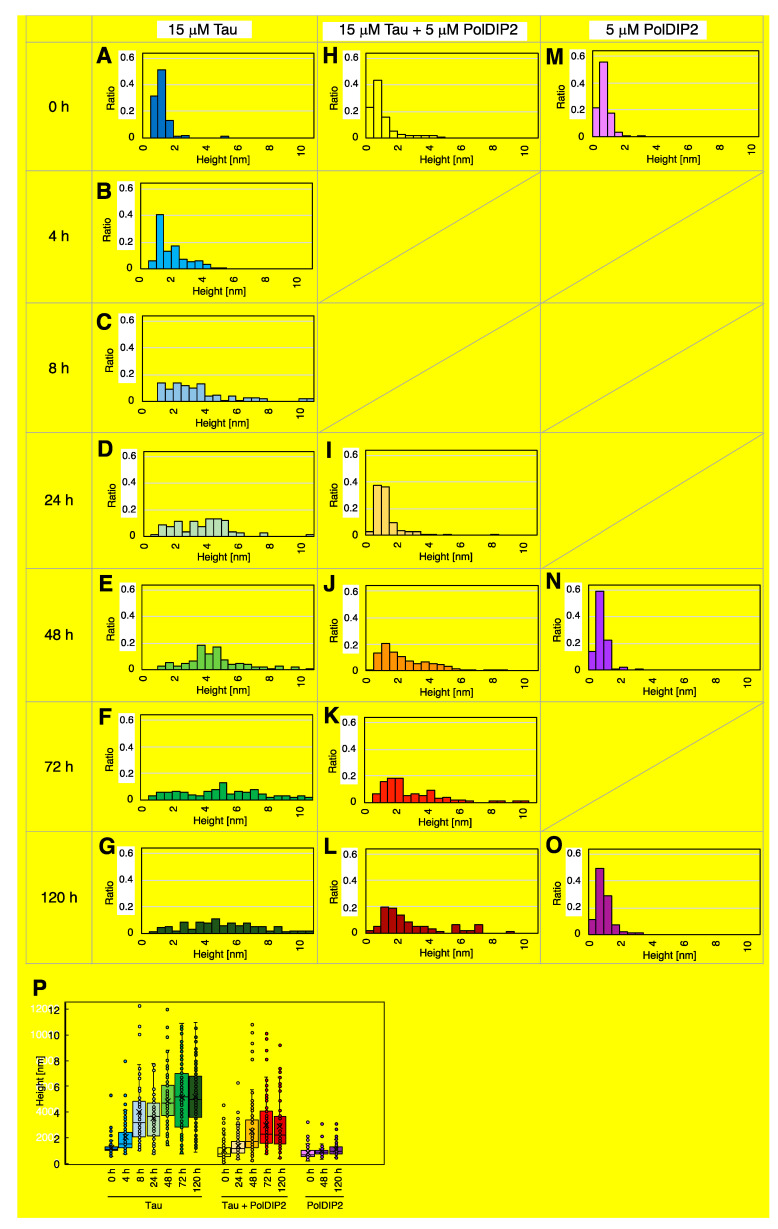
Tau forms globular oligomers in the presence of PolDIP2. (**A**–**C**) Height distribution of the fibrils or oligomers in samples with only Tau, measured in AFM cross sections at 0 h (**A**), 4 h (**B**), 8 h (**C**), 24 h (**D**), 48 h (**E**), 72 h (**F**), and 120 h (**G**); samples with PolDIP2 and Tau measured at 0 h (**H**), 24 h (**I**), 48 h (**J**), 72 h (**K**) and 120 h (**L**), and samples with only PolDIP2 measured at 0 h (**M**), 48 h (**N**) and 120 h (**O**). Distributions are presented by bar charts. (**P**) The statistical analysis of fibril and oligomer height shown in a box-and-whisker plot. The boxes represent the 25% to 75% percentiles of the data, whereas the whiskers represent the 5% to 95% percentiles of the data. The average height and median height are shown as a solid line (–) or cross (X) in the boxes.

To characterize the fibril structures formed by Tau in the presence and absence of PolDIP2, we performed a single oligomer/fibril analysis by AFM (Figure 3). AFM imaging of Tau fibril formation was carried out in parallel with the ThT monitoring of Tau aggregation kinetics. As expected, no Tau aggregation was detected at the start of the reaction, indicating that the purified recombinant Tau is initially monomeric when purified. Four hours after heparin addition, the Tau in solution form some “short fibrils” (defined as fibrils with 100–500 nm length; Figure 3D), while “long fibrils” (defined as fibrils with >500 nm length; Figure 3D) become the dominant form after 8 h of incubation, only becoming more abundant at later time points (Figure 3A). Image analysis of the Tau sample incubated for 48 h showed that 83% of the structures in the AMF imaging field (5 µm square size) represented “long fibrils”. Cross-section measurements of the Tau fibrils also showed fibril thickening over the course of the experiment (Figure 4). Initially, the height of the Tau fibril in cross-section was between 1 and 5 nm (Figure 4B,P), but at later time points substantially thicker fibrils (3 to 12 nm height) were observed (Figure 4C–G,P and Supplementary Figure S3). The thickening of the fibrillar threads is most likely the result of regular intertwining of multiple individual fibrils. Overall, we see that the Tau fibrils have a branched character with variable thickness (Supplementary Figure S3B).

In agreement with the dot-blot and ThT assays, AFM analysis revealed that Tau fibril formation was severely inhibited in the presence of PolDIP2 and “globular oligomers” (defined as round-shaped structures with <100 nm diameter and >2 nm height; Figure 3D) were mostly observed instead (Figure 3B). We did observe some rare “short fibrils” after a 48-h incubation period (Supplementary Figure S4); however, the absence of any “long fibrils” suggests that the specific Tau–PolDIP2 interaction inhibits Tau fibril formation (Figure 3B). After a prolonged incubation time (120 h), some Tau “long fibrils” were formed even in the presence of PolDIP2 (Figure 3B). This might indicate that the Tau–PolDIP2 interaction is reversible and transient, which is consistent with our size exclusion experiment (Figure 1). Despite the near absence of “long fibrils” in the presence of PolDIP2, we did observe accumulation of “globular oligomers”. The “globular oligomers” observed in the presence of PolDIP2 showed only moderate thickening of the aggregates over time (Figure 4H–L,P), with the average height of the “globular oligomers” increasing from 1.03 nm at 0 h incubation to 2.45 nm after 48 h incubation (Table 1).

**Figure 5:**
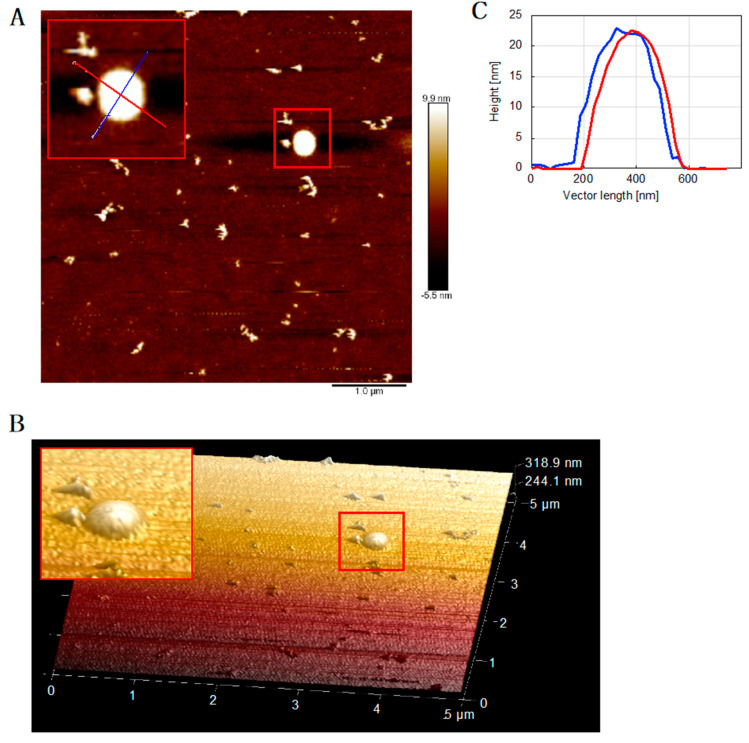
Formation of spherical complex “particulates” of Tau and PolDIP2. (**A**) AFM image of 15 µM Tau + 5 µM PolDIP2 at the 48-h reaction time (sample from the same reaction as in Figure 2A). (**B**) The 3D image was created based on the AFM image in Figure 5A. A spherical complex is highlighted with a red square. AFM cross sections are also inserted into Figure 5A shown by cross-sectional lines using the same color coding (**C**).

Strikingly, bigger round-shaped structures above 20 nm in height and >500 nm in diameter, termed “particulates” (Figure 3D) [24], were strictly observed in the AFM images obtained from samples that contained both Tau and PolDIP2 (Figure 5A,B; Supplementary Figure S6). These spherical structures (Figure 5C) are developed instead of Tau fibril formation, and their origin and structures should be examined in additional experiments.

When the PolDIP2 samples were analyzed in AFM, only “small oligomers” (defined as round-shaped structures with <2 nm height and <100 nm diameter.; Figure 3D) with an average height of 0.79 or 0.91 nm were observed at 0 or 48 h incubation, respectively (Figure 3C,D and Figure 4M,N; Table 1). This confirms that the “globular oligomers” of >2 nm in height are formed in the reactions that contained both Tau and PolDIP2, and suggests that PolDIP2 inhibits both the efficiency of Tau nucleation and fibril assembly, leading to a substantially slower Tau fibril formation. 

We saw an accumulation of “small oligomers” in the samples with 15 µM of PolDIP2 (Supplementary Figure S5), which are most likely responsible for the increased background signal in the ThT-based aggregation assays (Supplementary Figure S2B,C). During the first 4 h of incubation, we observed a moderate increase in the amount of these small PolDIP2 oligomers (Supplementary Figure S5A–C), but no further increase is seen at later time points (Supplementary Figure S5A,D). Importantly, there was no evidence of fibril formation in the samples with PolDIP2 alone (Supplementary Figure S5). Therefore, PolDIP2 aggregation, observed as increased ThT signal generation, does not hamper the Tau fibrillization analysis by AFM. 

## 4. Discussion

We show that recombinant human PolDIP2 can interact with Tau (Figure 1D). In agreement, in a previous study PolDIP2 was pulled down with an anti-FLAG antibody when FLAG-tagged Tau was exposed to rat brain lysates [6]. Interestingly, PolDIP2 was one of the rare interactors that associated specifically with the Tau monomer, but not with Tau fibrils, hinting to the possibility that PolDIP2 is a cellular regulator of Tau aggregation. In agreement, our ThT-based aggregation kinetic assays show that PolDIP2 is able to inhibit Tau aggregation (Figure 2A), which we further validated by using a Tau oligomer-specific (T22) antibody-based approach (Figure 2B) and AFM imaging (Figure 3). 

**Figure 6:**
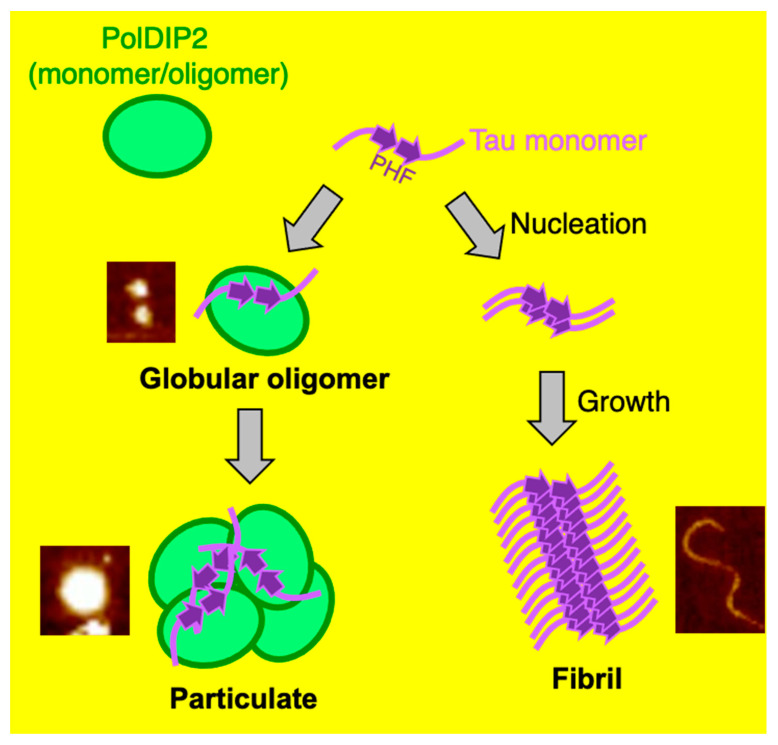
Summary of the PolDIP2-dependent inhibition of Tau aggregation. Tau is an unstructured protein, but in the presence of heparin, it forms oligomers that start fibril formation (nucleation), which then grow into longer fibrils (fibril growth). PolDIP2 interacts with the Tau monomer, and then it self-assembles into “globular oligomers”, which redirects its growth from linear to spherical assembly. The “globular oligomer” formation pathway is potentially behind the inhibition of the nucleation and fibril growth of Tau. Prolonged incubation of Tau with PolDIP2 leads to the formation of bigger round-shaped structures, termed “particulates”.

Based on our results, we propose a model where PolDIP2 interferes with Tau fibril formation. Without PolDIP2, Tau starts fibril formation (nucleation) in the presence of heparin, which gradually develop into longer fibrils (fibril growth) (Figure 6). PolDIP2 interacts specifically with the Tau monomer, forming “globular oligomers”, which likely do not interact with Tau oligomers or fibrils, therefore slowing Tau fibril formation. Prolonged incubation of Tau with PolDIP2 leads to the formation of big round-shaped “particulates” (Figure 6).

Previous studies identified specific antibodies or peptides that inhibit Tau aggregation through Tau interaction [16,17,18]. The Tau anti-aggregant peptides target two motifs, _275_VQIINK and _306_VQIVYK, which have the highest β-propensity at the beginning of the 2nd and 3rd repeat domains in Tau. Pir et al. showed that the hexapeptides VQPINK and VQPVYK decrease the rate of Tau aggregation in a concentration-dependent manner [17]. Interestingly, human PolDIP2 contains a comparable motif (_277_VQLRER) (Supplementary Figure S7), making it tempting to speculate that a similar mechanism might be behind the hexapeptide and PolDIP2-dependent inhibition of Tau aggregation. Parallel Tau aggregation and AMF experiments in the presence of either the hexapeptide VQPINK/VQPVYK and the PolDIP2-derived peptide (_277_VQLRER) could be used in future experiments to test this idea. It also would be interesting to test whether PolDIP2 can inhibit the aggregation of Tau, which is known to occur via the liquid–liquid phase separation (LLPS) process. In this process, the intrinsically disordered regions of Tau form local droplet structures via LLPS, which could also play a role in neuronal Tau aggregation [25,26].

### Limitations of This Study

Recent studies using cryo-EM [27] showed that heparin-induced Tau fibrils have a different structure from those found in AD brain; therefore, extrapolation of our findings toward diseases may be limited. Furthermore, in in vivo settings, phosphorylation of Tau plays a significant role in its self-aggregation [28]. As this study was performed with unphosphorylated Tau, it is possible that our in vitro data do not reflect in vivo Tau pathologies. Future research using ex vivo assays will need to validate the role of PolDIP2 in Tau aggregation inhibition.

## Figures and Tables

**Table 1:** Statistical data of the fibril height distributions for the samples presented in Figure 4.

Samples	Average Height (nm)	Median Height (nm)	Number
Tau, 0 h	1.21	1.12	101
Tau, 4 h	2.03	1.53	100
Tau, 8 h	4.04	3.15	100
Tau, 24 h	3.51	3.67	82
Tau, 48 h	4.82	4.71	109
Tau, 72 h	5.11	5.20	110
Tau, 120 h	5.20	4.97	119
Tau + PolDIP2, 0 h	1.03	0.71	99
Tau + PolDIP2, 24 h	1.36	1.17	104
Tau + PolDIP2, 48 h	2.45	1.70	141
Tau + PolDIP2, 72 h	2.96	2.27	98
Tau + PolDIP2, 120 h	2.88	2.17	96
PolDIP2, 0 h	0.79	0.65	89
PolDIP2, 48 h	0.91	0.89	94
PolDIP2, 120 h	1.06	0.96	100

## Data Availability

The data presented in this study are available on request from the corresponding authors.

## Supplementary Materials

The following are available online at https://www.mdpi.com/article/10.3390/ijms22115768/s1.
