# Supplementary material for: Human Polymerase δ-Interacting Protein 2 (PolDIP2) Inhibits the Formation of Human Tau Oligomers and Fibrils
Source: Int J Mol Sci. 2021 May 28;22(11):5768. doi: 10.3390/ijms22115768 (PMC8199196; doi:10.3390/ijms22115768)
Supplement: Supplementary file 1 [file ijms-22-05768-s001.zip › ijms-1200163-supplementary.pdf]

Supplementary Figures

Human polymerase δ-interacting protein 2 (PolDIP2) inhibits the formation of human Tau oligomers and fibrils.

Kazutoshi Kasho[1], Lukas Krasauskas[2], Vytautas Smirnovas[2], Gorazd Stojkovič[1], Ludmilla A. Morozova-Roche[1]* and Sjoerd Wanrooij[1]*

1. Department of Medical Biochemistry and Biophysics, Umeå University, Umeå, Sweden
2. Institute of Biotechnology, Life Sciences Center, Vilnius University, Vilnius, Lithuania.
*corresponding authors

Supplementary Figures

Supplementary Figure S1

A

```
TCGGACTCAGAAGTCAATCAAGAAGCTAAGCCAGAGGTCAAGCCAGAAGTCAAGCCTGAGACTC
ACATCAATTTAAAGGTGTCCGATGGATCTTCAGAGATCTTCTTCAAGATCAAAAAGACCACTCC
TTTAAGAAGGCTGATGGAAGCGTTCGCTAAAAGACAGGGTAAGGAAATGGACTCCTTAAGATTC
TTGTACGACGGTATTAGAATTCAAGCTGATCAGACCCCTGAAGATTTGGACATGGAGGATAACG
ATATTATTGAGGCTCACAGAGAACAGATTGGTGGGGCTGAGCCCCGCCAGGAGTTCGAAGTGAT
GGAAGATCACGCTGGGACGTACGGGTTGGGGGACAGGAAAGATCAGGGGGGCTACACCATGCAC
CAAGACCAAGAGGGTGACACGGACGCTGGCCTGAAAGAATCTCCCCTGCAGACCCCCACTGAGG
ACGGATCTGAGGAACCGGGCTCTGAAACCTCTGATGCTAAGAGCACTCCAACAGCGGAAGATGT
GACAGCACCCTTAGTGGATGAGGGAGCTCCCGGCAAGCAGGCTGCCGCGCAGCCCCACACGGAG
ATCCCAGAAGGAACCACAGCTGAAGAAGCAGGCATTGGAGACACCCCCAGCCTGGAAGACGAAG
CTGCTGGTCACGTGACCCAAGCTCGCATGGTCAGTAAAAGCAAAGACGGGACTGGAAGCGATGA
CAAAAAAGCCAAGGGGGCTGATGGTAAAACGAAGATCGCCACACCGCGGGGAGCAGCCCCTCCA
GGCCAGAAGGGCCAGGCCAACGCCACCAGGATTCCAGCAAAAACCCCGCCCGCTCCAAAGACAC
CACCCAGCTCTGGTGAACCTCCAAAATCAGGGGATCGCAGCGGCTACAGCAGCCCCGGCTCCCC
AGGCACTCCCGGCAGCCGCTCCCGCACCCCGTCCCTTCCAACCCCACCCACCCGGGAGCCCAAG
AAGGTGGCAGTGGTCCGTACTCCACCCAAGTCGCCGTCTTCCGCCAAGAGCCGCCTGCAGACAG
CCCCCGTGCCCATGCCAGACCTGAAGAATGTCAAGTCCAAGATCGGCTCCACTGAGAACCTGAA
GCACCAGCCGGGAGGCGGGAAGGTGCAGATAATTAATAAGAAGCTGGATCTTAGCAACGTCCAG
TCCAAGTGTGGCTCAAAGGATAATATCAAACACGTCCCGGGAGGCGGCAGTGTGCAAATAGTCT
ACAAACCAGTTGACCTGAGCAAGGTGACCTCCAAGTGTGGCTCATTAGGCAACATCCATCATAA
ACCAGGAGGTGGCCAGGTGGAAGTAAAATCTGAGAAGCTTGACTTCAAGGACAGAGTCCAGTCG
AAGATTGGGTCCCTGGACAATATCACCCACGTCCCTGGCGGAGGAAATAAAAAGATTGAAACCC
ACAAGCTGACCTTCCGCGAGAACGCCAAAGCCAAGACAGACCACGGGGCGGAGATCGTGTACAA
GTCGCCAGTGGTGTCTGGGGACACGTCTCCACGGCATCTCAGCAATGTCTCCTCCACCGGCAGC
ATCGACATGGTAGACTCGCCCCAGCTCGCCACGCTAGCTGACGAGGTGTCTGCCTCCCTGGCCA
AGCAGGGTTTGTGA
```

B

```
DSEVNQEAKPEVKPEVKPETHINLKVSDGSSEIFFKIKKTTPLRRLMEAFAKRQGKEMDSLRFL
YDGIRIQADQTPEDLDMEDNDIIEAHREQIGG|AEPRQEFEVMEDHAGTYGLGDRKDQGGYTMH
QDQEGDTDAGLKESPLQTPTEDGSEEPGSETSDAKSTPTAEDVTAPLVDEGAPGKQAAAQPHTE
IPEGTTAEEAGIGDTPSLEDEAAGHVTQARMVSKSKDGTGSDDKKAKGADGKTKIATPRGAAPP
GQKGQANATRIPAKTPPAPKTPPSSGEPPKSGDRSGYSSPGSPGTPGSRSRTPSLPTPPTREPK
KVAVVRTPPKSPSSAKSRLQTAPVPMPDLKNVKSKIGSTENLKHQPGGGKVQIINKKLDLSNVQ
SKCGSKDNIKHVPGGGSVQIVYKPVDLSKVTSKCGSLGNIHHKPGGGQVEVKSEKLDFKDRVQS
KIGSLDNITHVPGGGNKKIETHKLTFRENAKAKTDHGAEIVYKSPVVSGDTSPRHLSNVSSTGS
IDMVDSPQLATLADEVSASLAKQGL-
```

Supplementary Figure S1. SUMO-tagged Tau used in this study. (A) Nucleotide and (B) amino acid sequence of SUMO-Tau protein construct. Color legend: yellow; SUMO, magenta; ULP1 recognition sequence including cleavage site (l), sky blue; Tau (hTau441), and red; Stop codon.

Supplementary Figures

Supplementary Figure S2

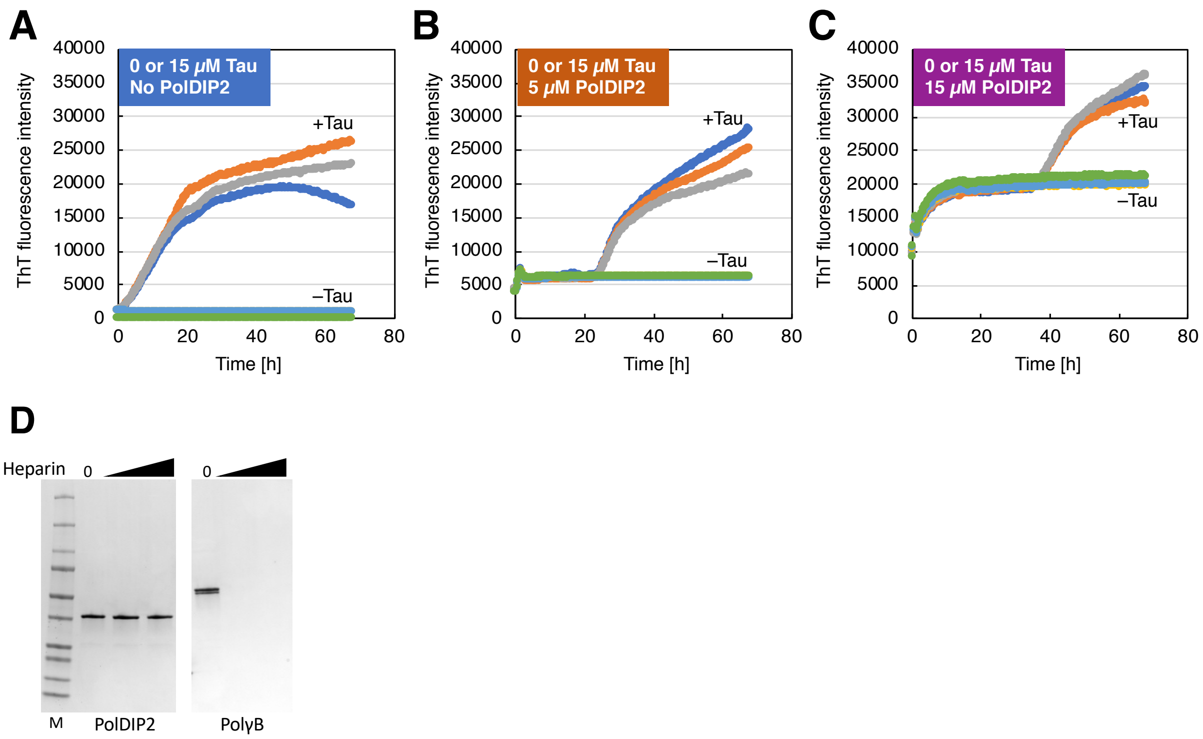

Supplementary Figure S2. PolDIP2-dependent inhibition of Tau aggregation, related to Figure 2A. 15 μM Tau in the presence of 8 μM heparin was incubated at 37°C in the absence (A) or presence of 5 μM (B) or 15 μM of PolDIP2 (C) and 50 μM of ThT. Reactions were done in 96-well microplates and fluorescent intensities were measured at 15 minutes intervals. Raw data was obtained from three replicates. (D) Interaction between PolDIP2 and heparin. 5 μM PolDIP2 was incubated with increasing concentrations of heparin sepharose beads (corresponding to heparin concentrations of 0, 8 and 40 μM) at 37°C for 30 min. The beads were pelleted and the unbound fraction (supernatant) was analyzed using SDS-PAGE.

Supplementary Figures

Supplementary Figure S3

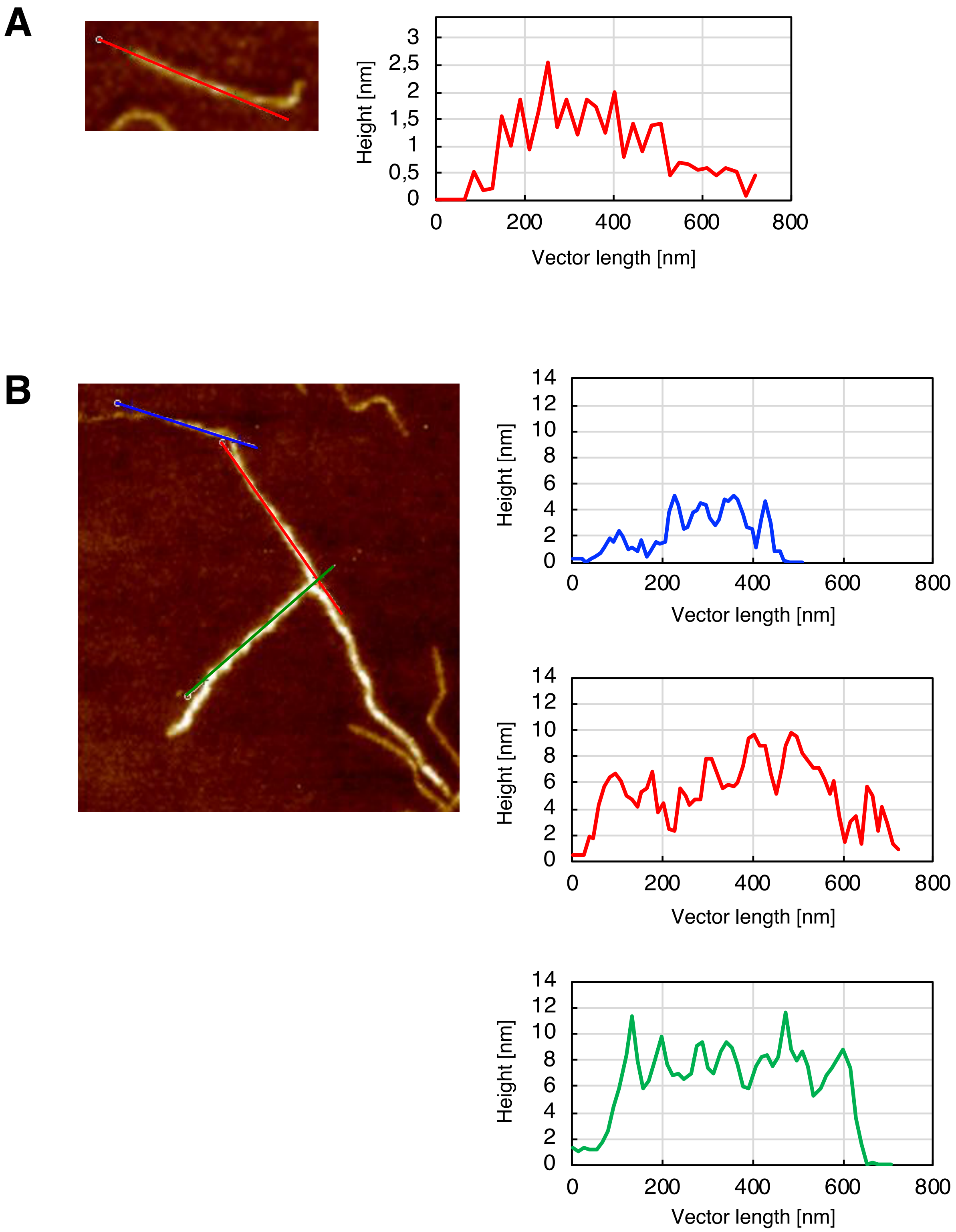

Supplementary Figure S3. The height of Tau “long fibrils” measured in AFM cross-sections, related to Figure 3A at 48 h incubation. The AFM cross-sections are shown in the panels next to the corresponding image. One thin fibril (A) and one thick, branched and intertwined fibril (B) were selected. The position of the cross-sections are shown by lines in the AFM images in the same colour-coding.

Supplementary Figures

Supplementary Figure S4

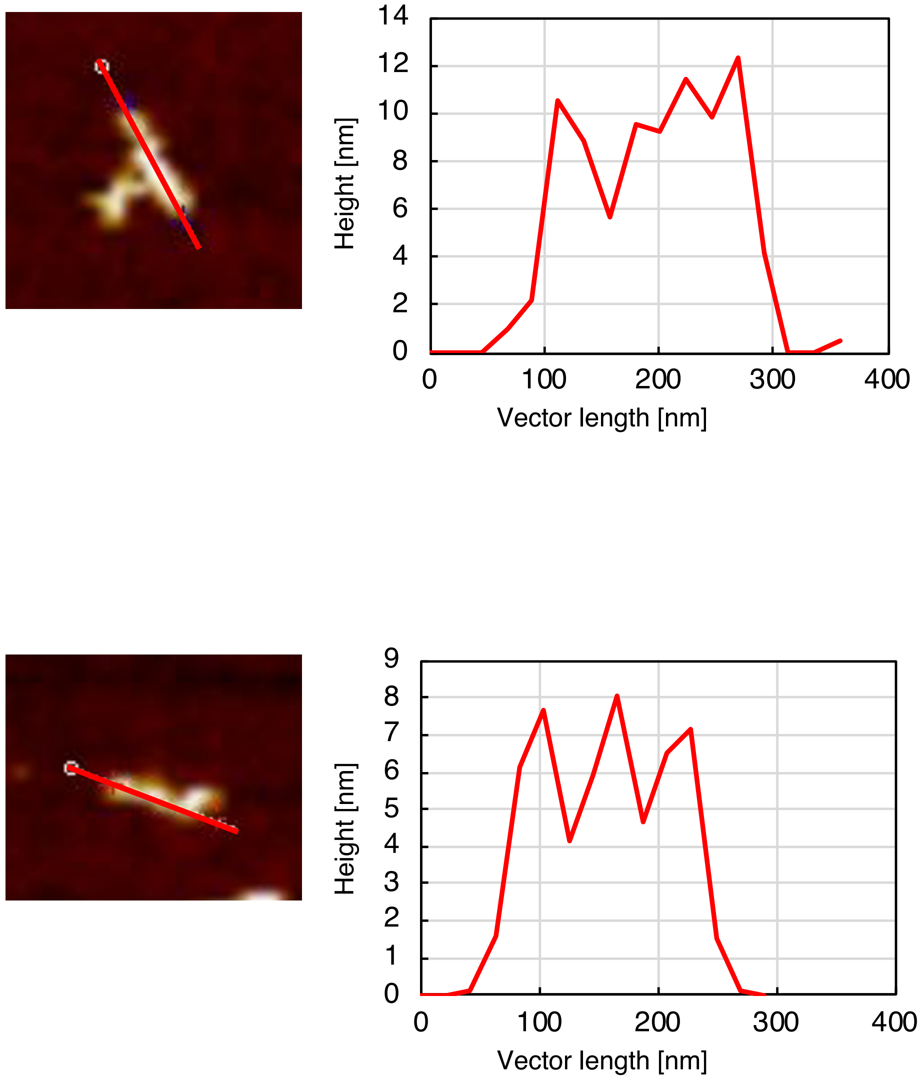

Supplementary Figure S4. The height of "short fibrils" of Tau incubated in the presence of PolDIP2 for 48 h and measured in AFM cross-section; related to Figure 3B. The AFM cross-sections are shown next to the corresponding images. Two fibrils were chosen from the images of Tau + PolDIP2 sample with 48 h incubation in Figure 3B. The position of the cross-sections are shown by red lines in the AFM images.

Supplementary Figures

Supplementary Figure S5

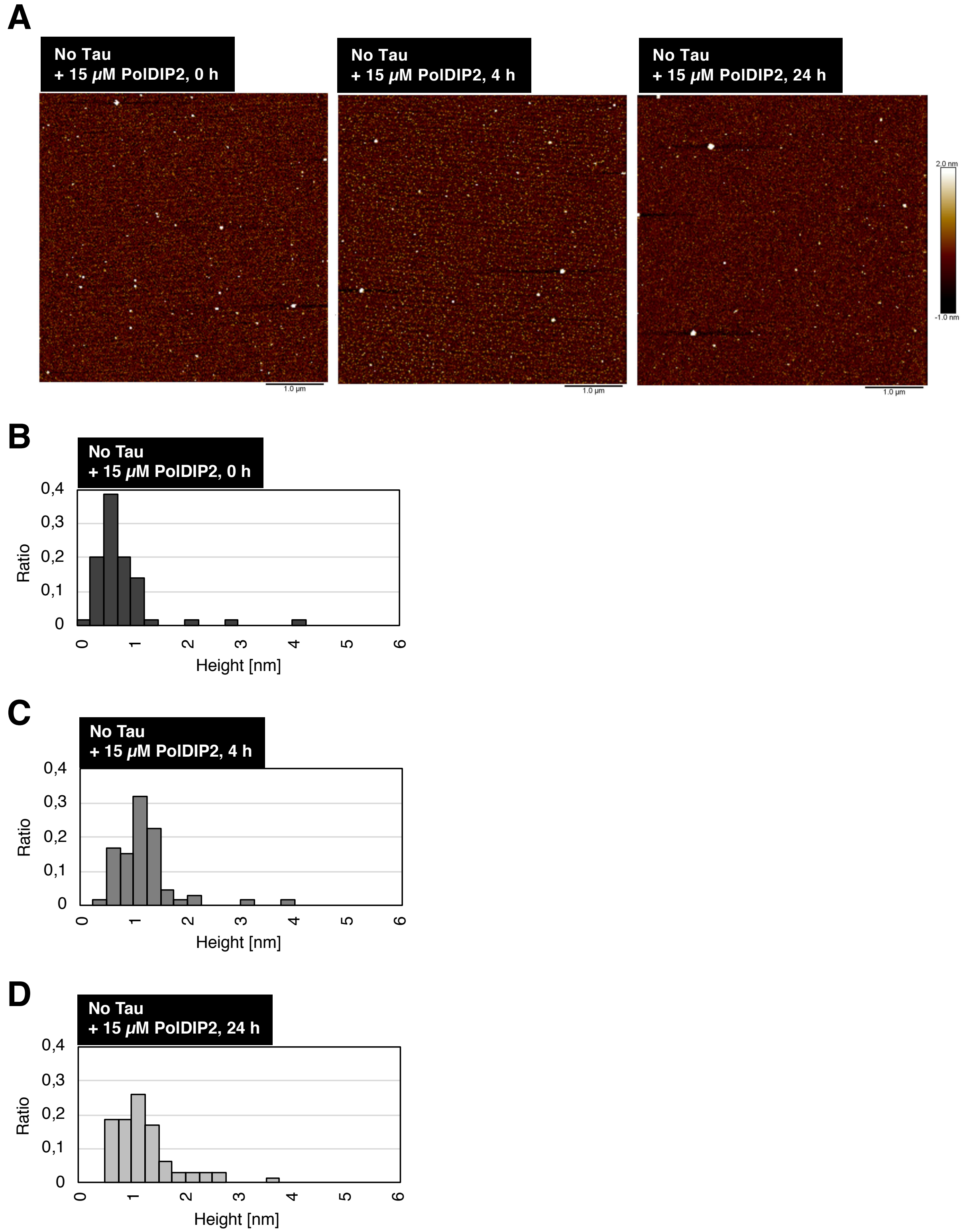

Supplementary Figure S5. Self-assembly of PolDIP2 into round-shaped particles during its incubation monitored by AFM. (A) AFM images of PolDIP2 (15 μM) samples at 0, 4 and 24 h. (B) The height distribution of round-shaped partciles in the samples with 15 μM PolDIP2 measured at 0 h (B), 4 h (C) or 24 h (D), analyzed using AFM cross-sections. Distributions are presented with bar charts.

Supplementary Figures

Supplementary Figure S6

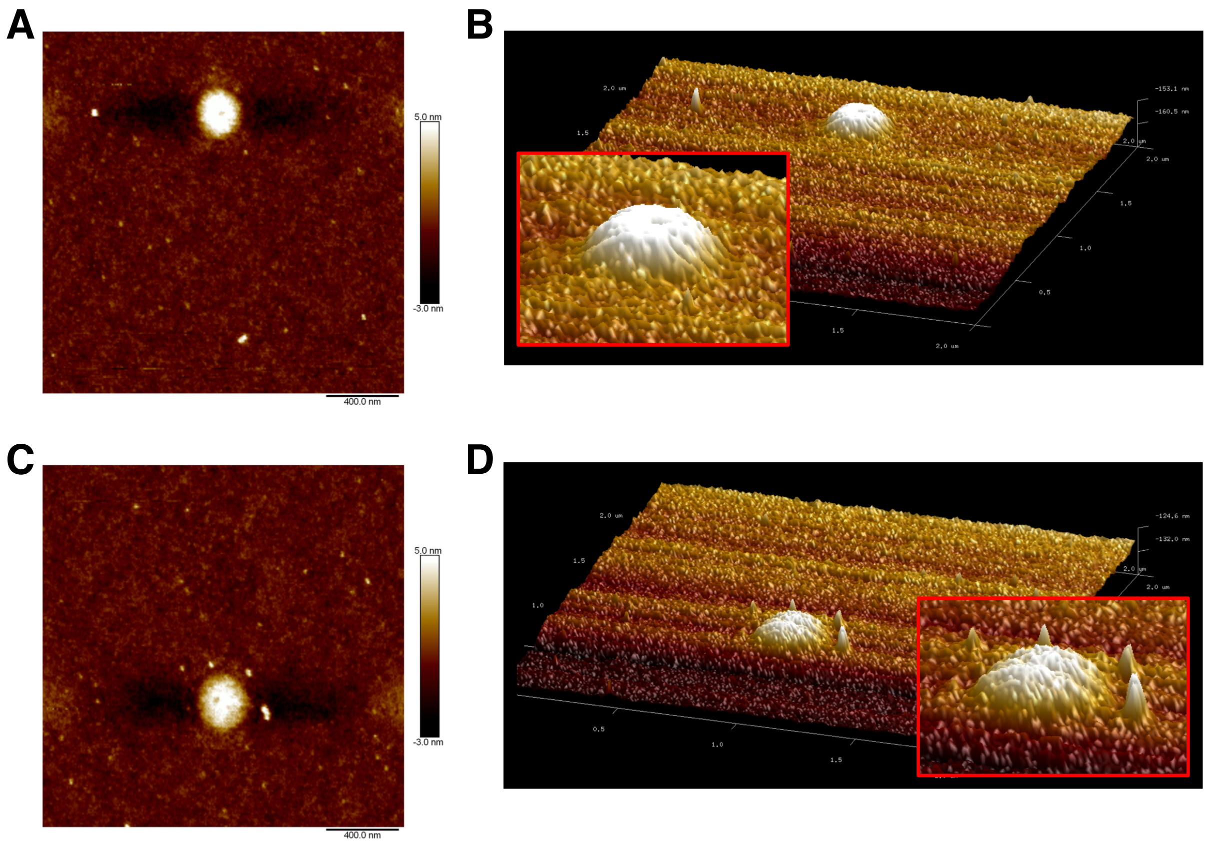

Supplementary Figure S6. "Particulates" in Tau + PolDIP2 sample incubated for 48 h, related to Figure 5. (A and C) Two different AFM images of 15 μM of Tau + 5 μM of PolDIP2 at 48 h reaction time (sample from the same reaction as Figure 2A). (B and D) The 3D AFM visualization of the same "particulate" as shown A and C. The "particulates" are highlighted using red squares.

Supplementary Figures

Supplementary Figure S7

```
PolDIP2_Human       MAACTARRALAVGSRWWSRSLTGARWPRPLCAAAGAGAFSPASTTTTRRHLSSRNRPEGK
TauPHF6_Human       ------------------------------------------------------------
TauPHF6*_Human      ------------------------------------------------------------

PolDIP2_Human       VLETVGVFEVPKQNGKYETGQLFLHSIFGYRGVVLFPWQARLYDRDVASAAPEKAENPAG
TauPHF6_Human       ------------------------------------------------------------
TauPHF6*_Human      ------------------------------------------------------------

PolDIP2_Human       HGSKEVKGKTHTYYQVLIDARDCPHISQRSQTEAVTFLANHDDSRALYAIPGLDYVSHED
TauPHF6_Human       ------------------------------------------------------------
TauPHF6*_Human      ------------------------------------------------------------

PolDIP2_Human       ILPYTSTDQVPIQHELFERFLLYDQTKAPPFVARETLRAWQEKNHPWLELSDVHRETTEN
TauPHF6_Human       ------------------------------------------------------------
TauPHF6*_Human      ------------------------------------------------------------

PolDIP2_Human       IRVTVIPFYMGMREAQNSHVYWWRYCIRLENLDSDVVQLRERHWRIFSLSGTLETVRGRG
TauPHF6_Human       ------------------------------------VQIVYK------------------
TauPHF6*_Human      ------------------------------------VQIINK------------------
                                                        **:  :

PolDIP2_Human       VVGREPVLSKEQPAFQYSSHVSLQASSGHMWGTFRFERPDGSHFDVRIPPFSLESNKDEK
TauPHF6_Human       ------------------------------------------------------------
TauPHF6*_Human      ------------------------------------------------------------

PolDIP2_Human       TPPSGLHW    368
TauPHF6_Human       --------    6
TauPHF6*_Human      --------    6
```

Supplementary Figure S7. Sequence similarity between PolDIP2 and Tau. Amino acid sequence of PolDIP2 was compared with Tau PHF6 (VQIVYK) and PHF6* (VQIINK) motifs using protein multiple sequence alignment tool Clustal Omega (EMBL-EBI). Identical amino acids are shown with a star (*), and those with similar chemical property are indicated with double dots (:).
